# Who goes there? Social surveillance as a response to intergroup conflict in a primitive termite

**DOI:** 10.1098/rsbl.2020.0131

**Published:** 2020-07-29

**Authors:** Faye J. Thompson, Kingsley L. Hunt, Kallum Wright, Rebeca B. Rosengaus, Erin L. Cole, Graham Birch, Avery L. Maune, Michael A. Cant

**Affiliations:** 1 Centre for Ecology and Conservation, University of Exeter, Penryn Campus, Cornwall TR10 9FE, UK; 2 Department of Marine and Environmental Sciences, Northeastern University, Boston, MA 02115, USA

**Keywords:** intergroup conflict, social cohesion, social evolution, affiliative behaviour, *Zootermopsis angusticollis*

## Abstract

Intergroup conflict has been suggested as a major force shaping the evolution of social behaviour in animal groups. A long-standing hypothesis is that groups at risk of attack by rivals should become more socially cohesive, to increase resilience or protect against future attack. However, it is usually unclear how cohesive behaviours (such as grooming or social contacts) function in intergroup conflict. We performed an experiment in which we exposed young colonies of the dampwood termite, *Zootermopsis angusticollis*, to a rival colony while preventing physical combat with a permeable barrier. We measured social contacts, allogrooming and trophallaxis before, during and after exposure. Termites showed elevated rates of social contacts during exposure to a rival compared to the pre-exposure phase, but rates returned to pre-exposure levels after colonies were separated for 9 days. There was evidence of a delayed effect of conflict on worker trophallaxis. We suggest that social contacts during intergroup conflict function as a form of social surveillance, to check individual identity and assess colony resource holding potential. Intergroup conflict may increase social cohesion in both the short and the long term, improving the effectiveness of groups in competition.

## 1. Introduction

Classic research on the evolution of cooperation has focused on mechanisms (such as kin selection and policing) that operate within social groups [1]. More recently, however, evidence has accumulated from studies of human and non-human animal societies that interactions between groups can exert a strong influence on within-group social behaviour [2–4]. Theoretical models developed to investigate the origins of large-scale human cooperation show that sufficiently intense conflict between groups for resources (intergroup conflict) can select for altruism within groups [5,6]. In these models, intergroup conflict can, over many generations, select for genetic traits that amplify both between-group aggression and within-group cooperation [7].

A hypothesis linked to, but distinct from, these models is that groups exposed to conflict should evolve to respond on a behavioural timescale by becoming more coordinated or cohesive, to increase effectiveness or resilience in group competition [8–10]. This is a classic idea in sociological and political studies of human conflict (the ‘conflict-cohesion hypothesis’; [11,12]). In non-human animal societies, there is indeed evidence that exposure to rival groups leads to increased ‘social cohesion’, measured by affiliative behaviour such as allogrooming or other social contacts [9,13–15]. However, in some systems, intergroup encounters are associated with reduced affiliation or increased within-group aggression [16–17]. Thus, there are intriguing and contrasting findings about whether intergroup conflict promotes internal solidarity or exacerbates internal conflicts.

Two limitations of existing research are, first, in behavioural ecology (as in political science; [12]), the concept of ‘social cohesion’ is rarely defined explicitly. Behaviours used as markers of cohesion or classed as affiliative may serve different functions in conflict and be differentially up- or downregulated accordingly. For example, groups might increase allogrooming behaviour to reduce stress following conflict, or to encourage participation in future intergroup interactions [13,18]. Conversely, groups might reduce allogrooming in favour of behaviours that maintain group integrity or defences, such as identity checking, vigilance or patrolling. Second, most studies measure only short-term impacts of exposure to conflict, over minutes or hours. It is largely unknown whether there are lasting effects on social cohesion, days or weeks after exposure to conflict (but see [19]).

In this study, we test how exposure to rival groups influences measures of social cohesion in the primitive dampwood termite *Zootermopsis angusticollis*. We test (i) the impact of intergroup conflict on behaviours that might promote social cohesion and (ii) whether there are any lasting impacts of intergroup conflict, long after exposure to rival groups has ceased. In *Z. angusticollis*, intergroup conflict is a key aspect of life history because multiple colonies compete for a single limited resource (a log) in which to develop, feed and reproduce [20–22], and that they never leave. Colonies that come into contact often engage in lethal combat, during which reproductives (kings and queens) are particularly likely to be killed [23].

We experimentally exposed colonies to a rival group while preventing physical combat by means of a permeable barrier and measured behaviour before, during and after exposure. We predicted that exposure to a rival colony would result in increased rates of social contact and affiliative behaviour. We also tested whether these behavioural responses persist long after colonies had been separated.

## 2. Methods

### (a) Study colonies

Experiments were conducted at the University of Exeter's Centre for Ecology and Conservation (UK) between November and December 2018. Incipient colonies used in these experiments were bred from stock colonies collected under permit from Redwood Regional Park, California (37°48′40″ N, 122°09′17″ W). Incipient colonies were formed by pairing de-winged virgin male and female alates harvested from stock colonies during dispersal events [24,25]. At the start of the study, colonies were aged between 66 and 564 days post-establishment. Incipient colonies were housed in plastic Petri dishes (10 cm × 10 cm) containing pieces of silver birch (*Betula pendula*) wood into which termites could burrow, and damp, cellulose filter paper (cut to 10 cm × 7 cm). Colonies were kept in a controlled environment room set at 23°C and 85% humidity, in total darkness, and were sprayed with distilled water approximately twice per week to maintain a damp environment.

### (b) Experimental setup

**Figure 1.**
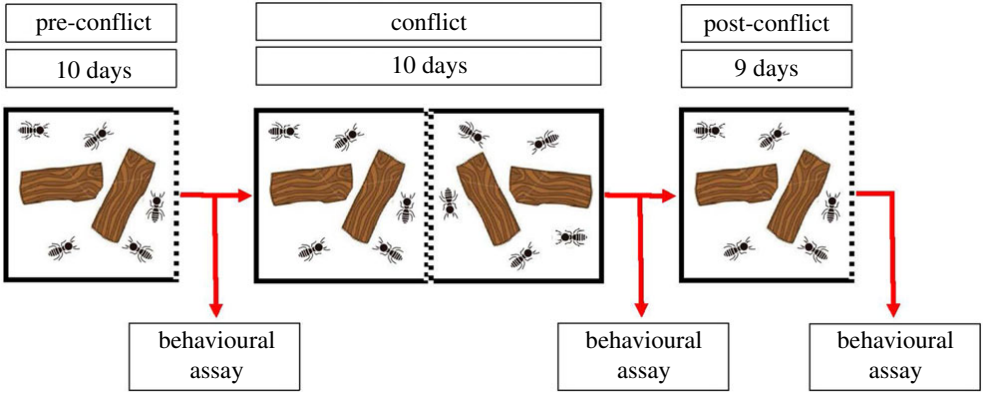
Schematic of the experimental design.

Thirty-three incipient colonies (mean ± s.e. colony size = 7.6 termites ± 0.65; range = 2–29; electronic supplementary material, table S1) were used in the experiment, which consisted of three phases (pre-conflict, conflict and post-conflict phases). Colonies consisted of a reproductive royal pair (king and queen), workers (the larval stage, also termed ‘pseudergates’, which perform worker tasks and have the capacity to differentiate into an alate [26]) and soldiers (but contained no secondary reproductives or reproductive soldiers; see electronic supplementary material, table S1 for colony compositions). In preparation for the conflict phase, one wall of each Petri dish was removed and replaced with a stainless steel mesh barrier with 2 mm holes (figure 1). This barrier prevented physical (potentially lethal) fighting while allowing for the detection of the rival colony via chemical and vibroacoustic cues and signals.

In the pre-conflict phase, colonies remained separate and undisturbed for 10 days, except for biweekly water spraying. On day 11, we extracted five individuals from each colony for behavioural observations. The king and queen were always extracted (if present). For colonies numbering fewer than five individuals, all colony members were used (electronic supplementary material, table S1). Extracted individuals were placed in a fresh 10 cm × 10 cm Petri dish (the observation arena) lined with clean filter paper. Termites were left to acclimatize for 15 min and then videoed for 10 min using a Sony HDR-PJ330 camera. Extracted individuals were filmed under red light using two 11 W light bulbs, before being returned to their original colonies.

At the start of the conflict phase (day 11), group size-matched pairs of colonies were placed adjacent to one another in contact along their mesh barrier and taped together. Pairs were matched for size to ensure that all colonies were exposed to a stimulus group of similar size to themselves. Pairs were left undisturbed for 10 days, except for bi-weekly water spraying. On day 22, we extracted 5 individuals from each colony, placed them in an observation arena, videoed them as before, then returned them to their original colonies.

At the start of the post-conflict phase (day 22), joined colonies were separated and, again, left undisturbed for 9 days (not 10 days, owing to a seasonal holiday), except for bi-weekly water spraying. On day 32, we conducted the final video recordings using methods described previously.

### (c) Behavioural and statistical analyses

Videos were analysed using BORIS version 7.4.5 [27]. We measured three behaviours that could be classed as affiliative: momentary social contacts (contacts lasting < 1 s, including antennation), allogrooming (more prolonged mandibular contact with body parts of the social partner) and trophallaxis (more prolonged mouth-to-mouth or mouth-to-anus contact). Counts (for social contacts) and duration (for allogrooming and trophallaxis) were scored for each individual that initiated the interaction in the observation arena. Across the three phases, we videoed 177 reproductives, 136 workers and 16 soldiers (electronic supplementary material, table S1). Since we did not have enough data in the pre-conflict phase to make meaningful comparisons of soldier behaviour across the phases, we excluded soldier behavioural data from our analyses.

Statistical analyses were performed in R version 3.6.0 [28]. For the analysis of social contacts, we fitted the number of social contacts as the response variable in a generalized linear mixed model (GLMM) using a Poisson error structure and a log link function. We included phase (pre-conflict, conflict, post-conflict), caste (reproductive, worker) and their interaction as fixed effects. We included the log(number of individuals in assay-1) as an offset term as a fixed effect to account for differences in the focal individual's opportunity to initiate social interactions. To control for differences in behaviour resulting from the presence of the queen [29], the king [30] or a soldier [30] in the colony, we included these variables as three additional fixed effects. Similarly, to control for the presence of a soldier in the observation arena [31], we included this variable as a fixed effect (electronic supplementary material, table S1). We fitted colony ID as a random effect and an observational level random effect to correct overdispersion of the response variable [32]. We fitted the model to 307 individuals (*N* = 102 pre-conflict; 102 conflict; 103 post-conflict) in 33 colonies [33].

For the analyses of allogrooming and trophallaxis, we fitted either the proportion of time spent allogrooming or engaging in trophallaxis as the response variable in a linear mixed model with a Gaussian error structure and identity link function. The response variable was logit transformed to ensure model residuals were normally distributed with homogeneous variance. We included phase, caste and their interaction as fixed effects, and an additional fixed effect of (number of individuals in assay-1) to account for differences in the focal individual's opportunity to initiate social interactions. As in the model of social contacts, we included whether there was the queen, the king or a soldier present in the colony, and whether there was a soldier present in the observation arena as fixed effects. We included colony ID as a random effect, and fitted each model to 307 individuals (*N* = 102 pre-conflict; 102 conflict; 103 post-conflict) in 33 colonies [33].

In each analysis, to assess the significance of each fixed effect we compared the likelihood ratio of the maximal model to that of the model without the fixed effect [34]. We removed non-significant interactions from our model to allow the main effects to be tested [35], but to avoid problems associated with stepwise model reduction we did not remove non-significant main effects [36,37].

## 3. Results

### (a) Rates of social contacts

**Figure 2.**
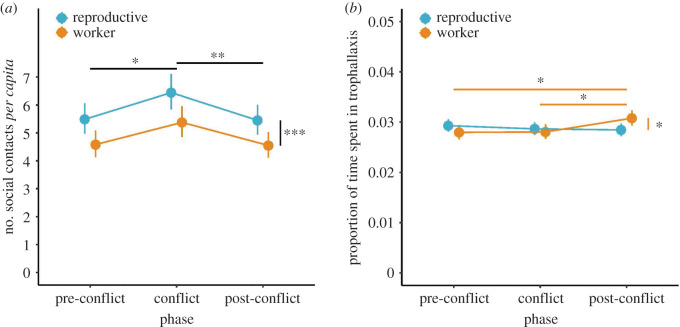
The effect of exposure to a rival group on (*a*) rates of social contacts, (*b*) the time spent in trophallaxis. In both panels, points show model-predicted means ± s.e. Asterisks refer to *post hoc* Tukey's all-pairwise comparisons of means. ***p < 0.001, **p < 0.01, *p < 0.05.

Individual termites initiated significantly more social contacts *per capita* at the end of the conflict phase than at the end of the pre-conflict or the post-conflict phase (GLMM, $\chi_{2}^{2}=10.47,$ *p* = 0.005; figure 2*a*; electronic supplementary material, table S2; *post hoc* Tukey's test, pre-conflict versus conflict: *β* ± s.e. = 0.16 ± 0.06, *z* = 2.63, *p* = 0.023; conflict versus post-conflict: *β* ± s.e. = −0.17 ± 0.05, *z* = −3.05, *p* = 0.006; electronic supplementary material, table S3). Overall, reproductives initiated significantly more social contacts than workers (*β* ± s.e. = −0.18 ± 0.05, $\chi_{1}^{2}=13.07,$ *p* < 0.001; figure 2*a*), but this effect was independent of phase ($\chi_{2}^{2}=0.37,$ *p* = 0.83). We observed fewer social contacts among individuals when the queen (*β* ± s.e. = −0.83 ± 0.25, $\chi_{1}^{2}=11.33,$ *p* < 0.001), the king (*β* ± s.e. = −0.53 ± 0.15, $\chi_{1}^{2}=12.88,$ *p* < 0.001), or a soldier was present in the colony (*β* ± s.e. = −0.50 ± 0.09, $\chi_{1}^{2}=27.12,$ *p* < 0.001).

### (b) Time allogrooming

There was no difference in the time that termites spent allogrooming across phases (LMM, $\chi_{2}^{2}=0.67,$ *p* = 0.72; electronic supplementary material, table S4), but overall workers spent significantly longer allogrooming than reproductives (*β* ± s.e. = 0.37 ± 0.07, $\chi_{1}^{2}=26.11,$ *p* < 0.001; interaction: $\chi_{2}^{2}=3.06,$ *p* = 0.22). Individuals spent longer allogrooming when the queen (*β* ± s.e. = 1.13 ± 0.27, $\chi_{1}^{2}=17.30,$ *p* < 0.001), the king (*β* ± s.e. = 0.71 ± 0.18, $\chi_{1}^{2}=14.69,$ *p* < 0.001), or a soldier was present in the colony (*β* ± s.e. = 0.46 ± 0.14, $\chi_{1}^{2}=11.30,$ *p* < 0.001).

### (c) Time in trophallaxis

There was a significant interaction between phase and caste (LMM, $\chi_{2}^{2}=7.76,$ *p* = 0.021; electronic supplementary material, table S5), which revealed that workers spent significantly longer in trophallaxis in the post-conflict phase compared to both the conflict and the pre-conflict phases (*post hoc* Tukey's test, conflict versus post-conflict: *β* ± s.e. = −0.10 ± 0.04, *t* = −2.54, *p* = 0.030; pre-conflict versus post-conflict: *β* ± s.e. = −0.10 ± 0.04, *t* = −2.45, *p* = 0.039; figure 2*b*; electronic supplementary material, table S6). Workers spent significantly longer in trophallaxis than reproductives in the post-conflict phase ( *β* ± s.e. = −0.08 ± 0.04, *t* = −2.19, *p* = 0.029; figure 2*b*; electronic supplementary material, table S6) but there was no change in trophallaxis by reproductives across phases.

## 4. Discussion

Rates of social contacts among colonymates increased during exposure to a rival colony, but returned to pre-exposure levels after colonies had been separated. We found no change in the duration of allogrooming but there was evidence of increased trophallaxis by workers in the post-conflict phase, long after conflict had ceased.

We suggest that our results reflect the different function of social behaviours in intergroup conflict. We hypothesise that elevated rates of social contact during exposure to a rival colony serve a ‘social surveillance’ function, to check the identity of individuals, or to assess absolute or relative resource holding potential. Frequent social contacts may be particularly important in the log environment in which *Z. angusticollis* lives, helping individuals to find and remain close to members of their own colony, and to detect enemies. By contrast, allogrooming and trophallaxis are less directly linked to the effectiveness of groups in combat, and so less likely to be expressed during exposure to rival groups. We suggest that ‘social cohesion’ should be defined and measured in terms of behaviours that plausibly increase the effectiveness or resilience of groups in conflict, rather than more generic ‘affiliative’ behaviours.

In most studies of real or simulated encounters, there is usually no information about the dynamics or durability of behavioural responses to conflict beyond a few hours (but see [19]). We found evidence that exposure to conflict had lasting effects on worker trophallaxis, 9 days after the stimulus was removed. This result is consistent with a shift in priorities or resource allocation after conflict, for example, via increased sharing of gut contents including symbionts that are essential for survival.

In other systems, there is great variety in the types of response that are assumed to represent social cohesion. Studies of social birds and mammals often use allogrooming or allopreening as measures of affiliation, and by implication, social cohesion [13]. In cichlid fish [9], affiliative behaviour takes the form of ‘bumps’ that resemble the contacts between termites in our study; momentary contacts have also been used as a measure of cohesion in ants [10], where they may also play a social surveillance role. How allogrooming or social contacts function to increase performance in conflict is usually unknown, although in vervet monkeys allogrooming by females appears to induce male participation in future bouts of aggression [16].

Our study adds to evidence that intergroup conflict shapes within-group behaviour, with effects that vary depending on the function of social interactions. Future research could usefully test how measures of cohesion affect group competitive ability, and the causes of variation in the durability of behavioural responses.

## Supplementary Material

Table of colony compositions and tables of statistical results

## Data accessibility

Data supporting this manuscript are available on Figshare: doi:10.6084/m9.figshare.11914395.
