# Supplementary material for: Who goes there? Social surveillance as a response to intergroup conflict in a primitive termite
Source: Biol Lett. 2020 Jul 29;16(7):20200131. doi: 10.1098/rsbl.2020.0131 (PMC7423038; doi:10.1098/rsbl.2020.0131)
Supplement: Table of colony compositions and tables of statistical results [file rsbl20200131supp1.docx]

**Electronic Supplementary Material**

**Who goes there? Social surveillance as a response to intergroup conflict in a primitive termite**

Faye J. Thompson^1^*, Kingsley L. Hunt^1^, Kallum Wright^1^, Rebeca B. Rosengaus^2^, Erin L. Cole^2^, Graham Birch^1^, Avery L. Maune^1^ & Michael A. Cant^1^*

*Corresponding authors:

Faye J. Thompson: [F.J.Thompson@exeter.ac.uk](about:blank)

Michael A. Cant: [M.A.Cant@exeter.ac.uk](about:blank)

1. Centre for Ecology and Conservation, University of Exeter, Penryn Campus, Cornwall TR10 9FE, UK.

2. Department of Marine and Environmental Sciences, Northeastern University, Boston, MA, USA

**Table S1.** Colony composition and assayed individuals during experimental phases. N = No; Y = Yes; *NA* indicates that census/assay data was not available for that phase.

Individuals extracted for behavioural assays: From 8 colonies (1 colony pre-conflict phase; 7 colonies conflict phase; 6 colonies post-conflict phase), a soldier was included among extracted individuals. Where we could not extract a total of 5 individuals without severely damaging the colony’s birch wood, we extracted as many individuals as possible (N=14 colonies pre-conflict phase; 12 colonies conflict phase; 8 colonies post-conflict phase). For colonies numbering fewer than 5 individuals all colony members were used (N=7 colonies pre-conflict phase; 9 colonies conflict phase; 11 colonies post-conflict phase). In total, we videoed 177 reproductives (63 pre-conflict phase; 58 conflict phase; 56 post-conflict phase), 136 workers (43 pre-conflict phase; 44 conflict phase; 49 post-conflict phase), and 16 soldiers (1 pre-conflict phase; 8 conflict phase; 7 post-conflict phase). Since we did not have enough data in the pre-conflict phase to make meaningful comparisons of soldier behaviour across phases, we excluded soldier behavioural data from our analyses.

**Table S2.** Model predicting the number of social contacts initiated by reproductive and worker termites in response to exposure to a rival colony. GLMM was fitted using a Poisson error structure and a log link function with an offset of log(number of individuals in assay-1), and with colony ID and an observation level term as random intercepts. N = 307 individuals (102 individuals in pre-conflict phase; 102 individuals in conflict phase; 103 individuals in post-conflict phase) in 33 colonies. Significant terms are given in bold.

| **Fixed effect** |  | | | **β** | **SE** | **χ^2^** | **p** |
| --- | --- | --- | --- | --- | --- | --- | --- |
| Intercept |  | | | 3.17 | 0.28 |  |  |
| **Phase** | Pre-conflict | | | 0.00 | 0.00 | 10.47 | **0.005** |
|  | Conflict | | | 0.16 | 0.06 |  |  |
|  | Post-conflict | | | -0.01 | 0.06 |  |  |
| **Caste** | Reproductive | | | 0.00 | 0.00 | 13.07 | **<0.001** |
|  | Worker | | | -0.18 | 0.05 |  |  |
| **Queen present** | No | | | 0.00 | 0.00 | 11.33 | **<0.001** |
|  | Yes | | | -0.83 | 0.25 |  |  |
| **King present** | No | | | 0.00 | 0.00 | 12.88 | **<0.001** |
|  | Yes | | | -0.53 | 0.15 |  |  |
| **Soldier present** | No | | | 0.00 | 0.00 | 27.12 | **<0.001** |
|  | Yes | | | -0.50 | 0.09 |  |  |
| Soldier in assay arena | No | | | 0.00 | 0.00 | 0.12 | 0.73 |
|  | Yes | | | -0.04 | 0.13 |  |  |
| Phase x Caste | Pre-conflict x Reproductive | | | 0.00 | 0.00 | 0.37 | 0.83 |
|  | Conflict x Reproductive | | | 0.18 | 0.07 |  |  |
|  | Post-conflict x Reproductive | | | -0.01 | 0.07 |  |  |
|  | Pre-conflict x Worker | | | -0.16 | 0.09 |  |  |
|  | Conflict x Worker | | | -0.06 | 0.11 |  |  |
|  | Post-conflict x Worker | | | -0.01 | 0.11 |  |  |
| **Random effect** | **Variance** | **SD** |  | | |  |  |
| Colony ID | 0.24 | 0.49 |  | | |  |  |
| Observation level term | 0.06 | 0.25 |  |  |  |  |  |

**Table S3.** Post-hoc multiple comparison of means using Tukey’s all-pairwise comparisons to determine differences in the number of social contacts across experimental phases. Original GLMM was fitted using a Poisson error structure and a log link function with an offset of log(number of individuals in assay-1), and with colony ID as a random intercept. N = 307 individuals (102 individuals in pre-conflict phase; 102 individuals in Conflict phase; 103 individuals in post-conflict phase) in 33 colonies. Significant post-hoc comparisons are given in bold.

| **Fixed effect** | **β** | **SE** | **z** | **p** |
| --- | --- | --- | --- | --- |
| **Pre-conflict versus Conflict** | 0.16 | 0.06 | 2.63 | **0.023** |
| **Conflict versus Post-conflict** | -0.17 | 0.05 | -3.05 | **0.006** |
| Pre-Conflict versus Post-conflict | -0.01 | 0.06 | -0.12 | 0.99 |

**Table S4.** Model predicting the proportion of time spent allogrooming by reproductive and worker termites in response to exposure to a rival colony. LMM was fitted using a Gaussian error structure and an identity link function, and with colony ID as a random intercept. N = 307 individuals (102 individuals in pre-conflict phase; 102 individuals in conflict phase; 103 individuals in post-conflict phase) in 33 colonies. Significant terms are given in bold.

| **Fixed effect** | | | |  | **β** | **SE** | **χ^2^** | **p** |
| --- | --- | --- | --- | --- | --- | --- | --- | --- |
| Intercept | | | |  | -4.91 | 0.33 |  |  |
| Phase | | | | Pre-conflict | 0.00 | 0.00 | 0.67 | 0.72 |
|  | | | | Conflict | 0.04 | 0.08 |  |  |
|  | | | | Post-conflict | -0.02 | 0.08 |  |  |
| **Caste** | | | | Reproductive | 0.00 | 0.00 | 26.11 | **<0.001** |
|  | | | | Worker | 0.37 | 0.07 |  |  |
| **Queen present** | | | | No | 0.00 | 0.00 | 17.30 | **<0.001** |
|  | | | | Yes | 1.13 | 0.27 |  |  |
| **King present** | | | | No | 0.00 | 0.00 | 14.69 | **<0.001** |
|  | | | | Yes | 0.71 | 0.18 |  |  |
| **Soldier present** | | | | No | 0.00 | 0.00 | 11.30 | **<0.001** |
|  | | | | Yes | 0.46 | 0.14 |  |  |
| Soldier in assay arena | | | | No | 0.00 | 0.00 | 2.90 | 0.08 |
|  | | | | Yes | 0.30 | 0.18 |  |  |
| **Number others in assay arena** | | | |  | -0.13 | 0.06 | 4.34 | **0.04** |
| Phase x Caste | | | | Pre-conflict x Reproductive | 0.00 | 0.00 | 3.06 | 0.22 |
|  | | | | Conflict x Reproductive | 0.12 | 0.10 |  |  |
|  | | | | Post-conflict x Reproductive | -0.03 | 0.10 |  |  |
|  | | | | Pre-conflict x Worker | 0.43 | 0.11 |  |  |
|  | | | | Conflict x Worker | -0.21 | 0.15 |  |  |
|  | | | | Post-conflict x Worker | 0.01 | 0.14 |  |  |
| **Random effect** | **Variance** | **SD** |  | | | |  |  |
| Colony ID | 0.18 | 0.43 |  | | | |  |  |

**Table S5.** Model predicting the proportion of time spent in trophallaxis by reproductive and worker termites in response to exposure to a rival colony. LMM was fitted using a Gaussian error structure and an identity link function, and with colony ID as a random intercept. N = 307 individuals (102 individuals in pre-conflict phase; 102 individuals in conflict phase; 103 individuals in post-conflict phase) in 33 colonies. Significant terms are given in bold.

| **Fixed effect** | | | |  | **β** | **SE** | **χ^2^** | **p** |
| --- | --- | --- | --- | --- | --- | --- | --- | --- |
| Intercept | | | |  | -3.53 | 0.11 |  |  |
| Phase | | | | Pre-conflict | 0.00 | 0.00 |  |  |
|  | | | | Conflict | -0.02 | 0.03 |  |  |
|  | | | | Post-conflict | -0.03 | 0.03 |  |  |
| Caste | | | | Reproductive | 0.00 | 0.00 |  |  |
|  | | | | Worker | -0.05 | 0.04 |  |  |
| Queen present | | | | No | 0.00 | 0.00 | 0.39 | 0.53 |
|  | | | | Yes | -0.06 | 0.08 |  |  |
| King present | | | | No | 0.00 | 0.00 | 1.88 | 0.17 |
|  | | | | Yes | 0.09 | 0.06 |  |  |
| Soldier present | | | | No | 0.00 | 0.00 | 0.10 | 0.75 |
|  | | | | Yes | -0.01 | 0.05 |  |  |
| Soldier in assay arena | | | | No | 0.00 | 0.00 | 0.03 | 0.86 |
|  | | | | Yes | -0.01 | 0.06 |  |  |
| Number others in assay arena | | | |  | 0.02 | 0.02 | 0.60 | 0.44 |
| **Phase x Caste** | | | | Pre-conflict x Reproductive | 0.00 | 0.00 | 7.76 | **0.021** |
|  | | | | Conflict x Reproductive | -0.02 | 0.03 |  |  |
|  | | | | Post-conflict x Reproductive | -0.03 | 0.03 |  |  |
|  | | | | Pre-conflict x Worker | -0.05 | 0.04 |  |  |
|  | | | | Conflict x Worker | 0.03 | 0.05 |  |  |
|  | | | | Post-conflict x Worker | 0.13 | 0.05 |  |  |
| **Random effect** | **Variance** | **SD** |  | | | |  |  |
| Colony ID | 0.02 | 0.13 |  | | | |  |  |

**Table S6.** Post-hoc multiple comparison of means using Tukey’s all-pairwise comparisons to determine differences in the proportion of time in trophallaxis for reproductive and worker termites across experimental phases. Original LMM was fitted using a Gaussian error structure and an identity link function, and with colony ID as a random intercept. N = 307 individuals (102 individuals in pre-conflict phase; 102 individuals in conflict phase; 103 individuals in post-conflict phase) in 33 colonies. Significant post-hoc comparisons are given in bold.

|  | **Fixed effect** | **β** | **SE** | **t** | **p** |
| --- | --- | --- | --- | --- | --- |
| Reproductives | Pre-conflict versus Conflict | 0.02 | 0.03 | 0.68 | 0.77 |
|  | Conflict versus Post-conflict | 0.01 | 0.03 | 0.22 | 0.97 |
|  | Pre-Conflict versus Post-conflict | 0.03 | 0.03 | 0.90 | 0.64 |
| Workers | Pre-conflict versus Conflict | -0.003 | 0.04 | -0.07 | 0.99 |
|  | **Conflict versus Post-conflict** | -0.10 | 0.04 | -2.54 | **0.030** |
|  | **Pre-Conflict versus Post-conflict** | -0.10 | 0.04 | -2.45 | **0.039** |
| Pre-conflict | Reproductives versus Workers | 0.05 | 0.04 | 1.24 | 0.21 |
| Conflict | Reproductives versus Workers | 0.02 | 0.04 | 0.58 | 0.56 |
| **Post-conflict** | **Reproductives versus Workers** | -0.08 | 0.04 | -2.19 | **0.029** |
